# Post-pandemic e-learning: a pre-protocol to assess the integration of mobile VR and its effect on VARK learning styles

**DOI:** 10.12688/f1000research.73311.1

**Published:** 2021-11-02

**Authors:** Shahida Raihan Manzoor, Wan-Noorshahida Mohd-Isa, Khairi Shazwan Dollmat

**Affiliations:** 1 Faculty of Computing and Informatics, Multimedia University, Cyberjaya, Selangor, 63100, Malaysia

**Keywords:** E-Learning, Mobile VR, Learning Style, Post- pandemic Education

## Abstract

**Background: **The Covid-19 pandemic has resulted in an abrupt but accelerated shift to e-learning worldwide. Education in a post-pandemic world has to amalgamate the advantages of e-learning with important pedagogical goals associated with in-person teaching. Although various advanced technologies are present at our fingertips today, we are still unable to use their full potential in teaching and learning. In this regard, mobile VR technology is both cost-efficient, versatile and engaging for students. Developing countries have more smartphone users than developed countries, implying that developing countries, like Malaysia, should utilize mobile or cellphones more significantly. With that in mind, we propose here a pre-protocol to investigate learner motivation and levels of engagement for e-learning with smartphone-integrated VR, based on their VARK (Visual, Auditory, Read/Write, Kinesthetic) learning styles.

**Proposed methodology: **This study intends to use a minimum sample of 30 students from the same age group under the K-12 (particularly grade 9-12) belonging to STEM curriculum. The Google Cardboard VR set will be used as the prime technology for its affordability, easy build feature and variety of available vendors. A mixed-method (survey and activity log/tracking) for data collection is suggested to find the degree of engagement and motivation of the learners’ learning in the mobile VR-assisted e-learning context. The students will be taught a topic using the mobile VR and then be assessed through simple classroom quizzes to assess how well they grasped the concept. The data collected through activity logs (while teaching the topic in mobile VR) and questionnaires will be mapped to each individual learner and organized in a data repository. Further visualization, analysis and investigation will be performed using Smart PLS, Python or R language.

**Conclusions: **The study aims to provide context for smartphone and software companies to develop technologies that could facilitate learner engagement during the post-pandemic state.

## Introduction

Since the Covid-19 pandemic broke out in 2020, e-learning has become a new standard worldwide. A staggering number of educational institution closures took place in 192 countries, as reported by the International Labour Organization (ILO), resulting in 91.4% of enrolled learners being affected. ^1^ To tackle these circumstances, a number of educational institutions had been forced to abruptly adopt full online learning measures.

One of the sectors that is being heavily affected by these measures is the education sector, particularly school students. According to a survey conducted by RAND Corporation, teachers of virtual classrooms need more overall instructional support compared to teachers teaching in physical classrooms, in terms of tailoring content, engaging students, academically advancing them and measuring their progress. ^2^ Access to digital devices and the internet was a concern too. ^2^

Numerous educators often use smartphones for e-learning in many parts of the world. Because of the perceived utility of mobile phones, learners in such contexts may suffice without a computer to access e-learning materials. ^3^^,^^4^ Yet, uses of mobiles for learning are mostly limited among tertiary-level students. According to Darko-Adjei, ^5^ learners were not prepared for the integration of mobile phones in the learning context, especially in Malaysian schools. Moreover, there is a lack of awareness of its potential power of innovation in the education system. ^5^

In addition to that, traditional e-learning tends to face challenges in retaining learner motivation and keeping them engaged, as well as certain limitations in explaining abstract scientific concepts to different types of learners. According to Stovall, ^6^ in the context of web-based learning systems, “a student’s degree of engagement in educational learning is lower than that in traditional education systems”. As cited in Hartnetter, ^17^ e-learners are frequently expected to be further naturally motivated as the learning environment depends heavily on their interest, self-drive, and intrinsic motivation to evoke student engagement.

In reality, some regard the technology used as intrinsically motivating since it offers a range of properties acknowledged as essential in the development of intrinsic motivation, notably, novelty, challenge, fantasy and curiosity. ^7^ The study proposed here intends to investigate learners’ motivation and levels of engagement for e-learning with smartphone-integrated VR for the K-12 student cohort in Malaysian schools. Accordingly, it will provide recommendations for smartphone and software companies to develop technologies integrating various learning styles that could facilitate learning engagement during the post-pandemic state.

## Literature review

### VARK learning style

The learning styles of an individual pertain to his or her ability to effectively understand and absorb information. ^8^ In terms of student-centric learning, it is essential to accommodate all students with different learning preferences. Many teachers believe that teaching according to individual style can help improve learners’ performance. ^24^ Mirza and Khurshid ^8^ identified the learning modalities of health professional students and suggested that student motivation is positively enhanced when students recognized their learning styles. The study by Good *et al.* ^24^ also suggested that VARK (Visual, Aural, Read/write, Kinesthetic) is a significant teaching tool and also helps enhance performance.

Mirza and Khurshid ^8^ have stated that VARK (Visual, Auditory, Reader/Writer and Kinesthetic) is the most accepted teaching model that categorizes learners with respect to their sensory characteristics. It is one of the earliest and most popular learner style tools developed by Fleming and Mills. ^19^ According to these researchers, the success of the model stems from its authenticity, usability, and the array of learning resources available to complement it.

### E-learning and its effectiveness

The term e-learning or electronic learning originated in the mid-1990s when the Internet began to gather momentum. ^20^ The two core elements of e-learning include computer-based learning as well as web-based learning. ^9^

**Figure 1.**
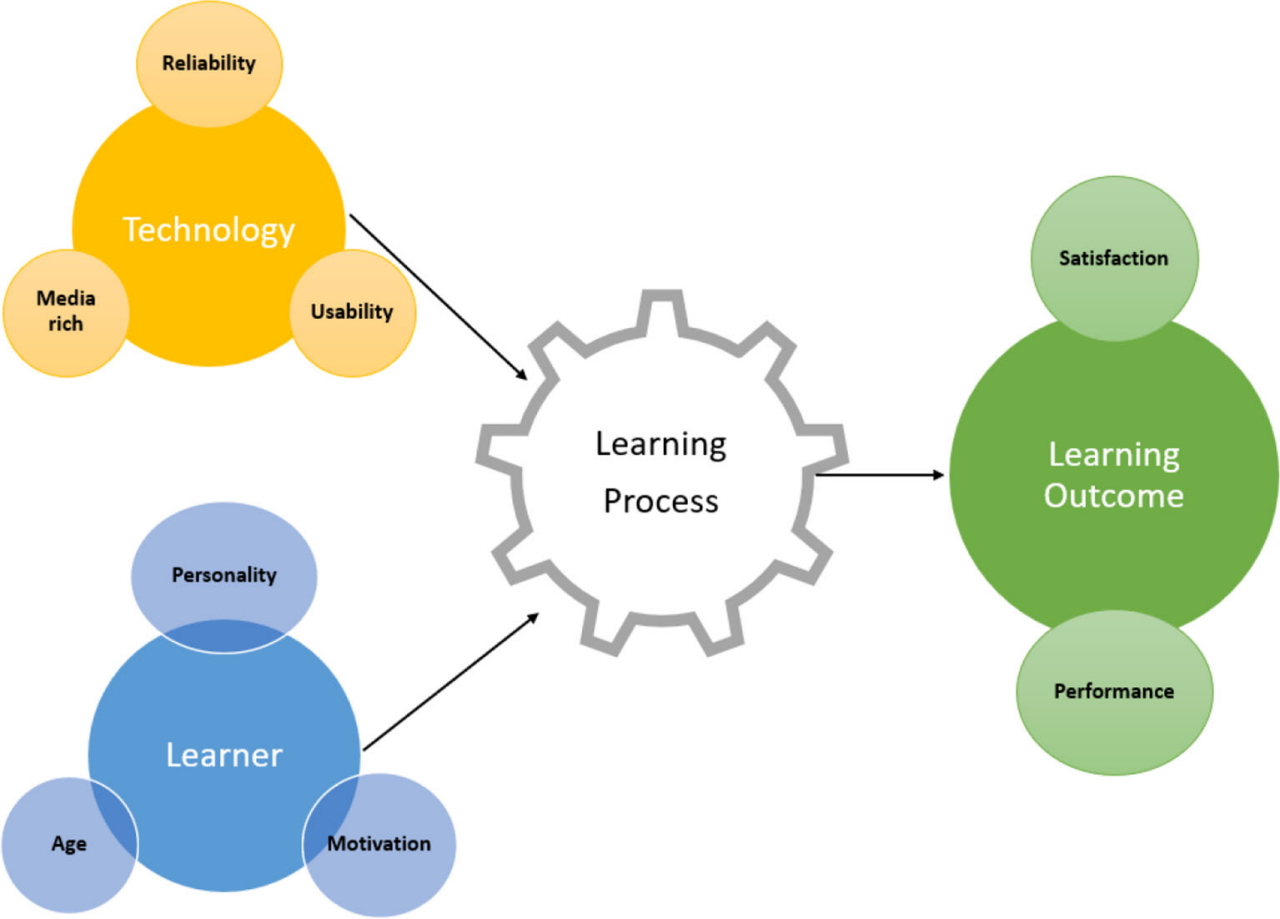
A model of e-learning influences and its effectiveness.

Researchers have developed a number of models to guide e-learning research and e-learning effectiveness. By studying and synthesizing previous models of e-learning research from management, information systems, education, sociology and psychology, Johnson and Brown ^10^ concluded that e-learning outcomes are influenced by multiple processes and inputs. They proposed a model to summarize the literature consisting of five inputs: organizational context, technology, design/pedagogy, instructions and trainee or learner traits. The study by Johnson and Brown focuses more on *technology* as an input and influencing factor in the learning process. The sub focuses under *technology* are reliability, usefulness, ease of use and media richness ( Figure 1).

### Mobile learning

**Figure 2.**
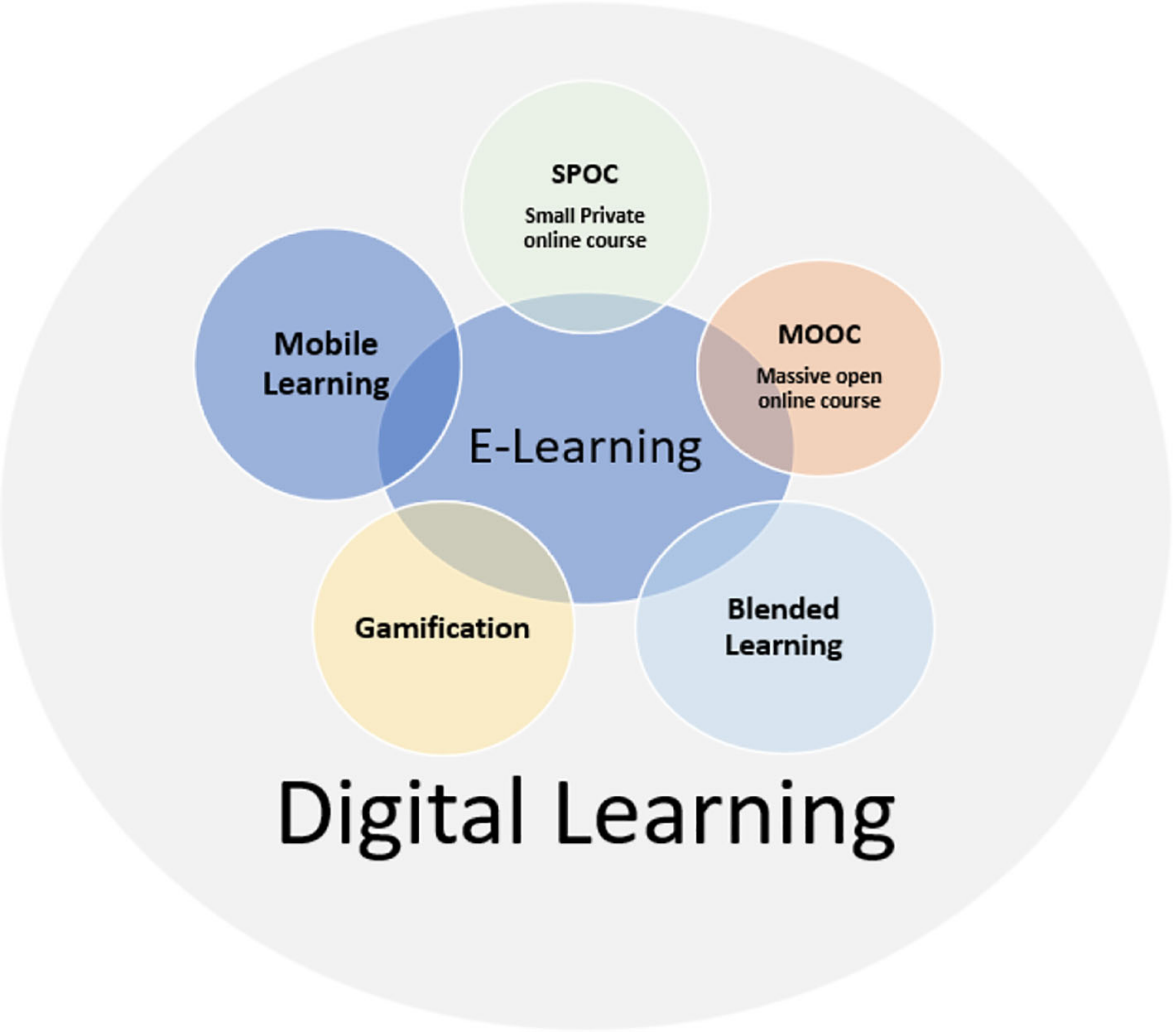
Relationship between e-learning (electronic learning), m-learning (mobile learning), and d-learning (digital learning).

Mobile learning is an integral part of e-learning. As cited by Basak *et al.*, ^9^ Behera stated that mobile learning or m-learning is correlated with mobile computing and e-learning. According to Sanchez-Prieto *et al.*, ^18^ mobile learning is a learning environment that is closely related to e-learning, belonging to a separate typology, where the process of teaching, as well as learning, would have a digital dimension ( Figure 2).

### VR in education and learner engagement

Virtual reality (VR) is a computer-generated rendering of a 3D world or image that can be communicated in a relatively natural or tactile manner by a human wearing special electronic devices (headgear with an incorporated display inside or accessories equipped with sensors). VR provides 3D digital worlds featuring sophisticated ways of interaction that can motivate students to understand more completely. Its truly interactive learning environment has the power to improve the learning process and skill acquisition. ^12^ Previous research has shown that the use of VR technologies will help to motivate the learning process by allowing students to experience ‘natural phenomena’ while being safe from the real-world consequences. ^12^ In other words, the incorporation of VR allows new educational opportunities to emerge.

Teachers frequently encounter issues concerning student engagement with teaching materials. ^22^ Higher levels of engagement with learning activities can be achieved if the virtual world is more interactive. For instance, debates are typically effective at involving learners in subjects needing critical thinking, ^21^ but they are less suitable for learning factual science subjects like physics or chemistry. VR may be particularly useful for those subjects where a spatial arrangement is important or there are dynamic changes.

VR can enhance engagement and improve retention, as students get to learn through experience, which is necessary for STEM subjects. VR also influences the students’ spatial ability for real-time visualization, which are crucial skills in the engineering field. Students can explore “the use of advanced holographic technologies to bring virtual 3D building components to life” [ 11, para 3]. The implications of VR require investment in resources and technical equipment. Hence there is a need for educational institutions to start with “cheaper alternatives and small mobile devices first” [ 11, para 5].

*VR and learner motivation*

Many professions, including medical science, engineering, architecture, product development, and geology, have used VR to study and visualize abstract concepts. ^25^ According to previous research, utilizing VR technology as a teaching tool enhances learners' grasp on the concept, test scores as well as learning motivation while lowering training costs and experimental risks. Employing VR in instruction can potentially increase student learning motivation and positively enhance student performance. ^26^ However, there is a paucity of studies on the impact of mobile VR on student motivation in STEM courses.

*Mobile VR*

Although VR has been available for a long time, the associated technology required to access it has been prohibitively expensive, heavy and high battery/power consumption. VR apps have been able to proliferate into the general market because of mobile VR headsets, which are essentially eyewear that can support a smartphone. ^13^

With a number of available ways to experiment with VR content, headsets, video and apps, this tool has the potential to become yet another method in an educator's repertoire for assisting learners in understanding critical theories and mastering a range of concepts. As smartphones with integrated video capability became widely available, the influence of what could be shared and generated in the classroom has improved significantly. VR technology is essentially the first phase leading to the advancement of interactive learning, ^1^ and mobile VR adds accessibility and cost-efficiency to that advancement.

## Proposed methodology

### Hypotheses and framework

Based on the above review of literature, the following hypotheses are proposed for this study:

H1: Visual learners have the highest level of engagement in a mobile VR environment.

H2: Auditory learners have the highest level of engagement in a mobile VR environment.

H3: Kinesthetic learners have the highest level of engagement in a mobile VR environment.

H4: Reader/Writer has the least engagement in a mobile VR environment.

H5: Visual learners have the highest level of motivation in a mobile VR environment.

H6: Auditory learners have the highest level of motivation in a mobile VR environment.

H7: Kinesthetic learners have the highest level of motivation in a mobile VR environment.

H8: Reader/Writer learners are least motivated to learn in a mobile VR environment.

**Figure 3.**
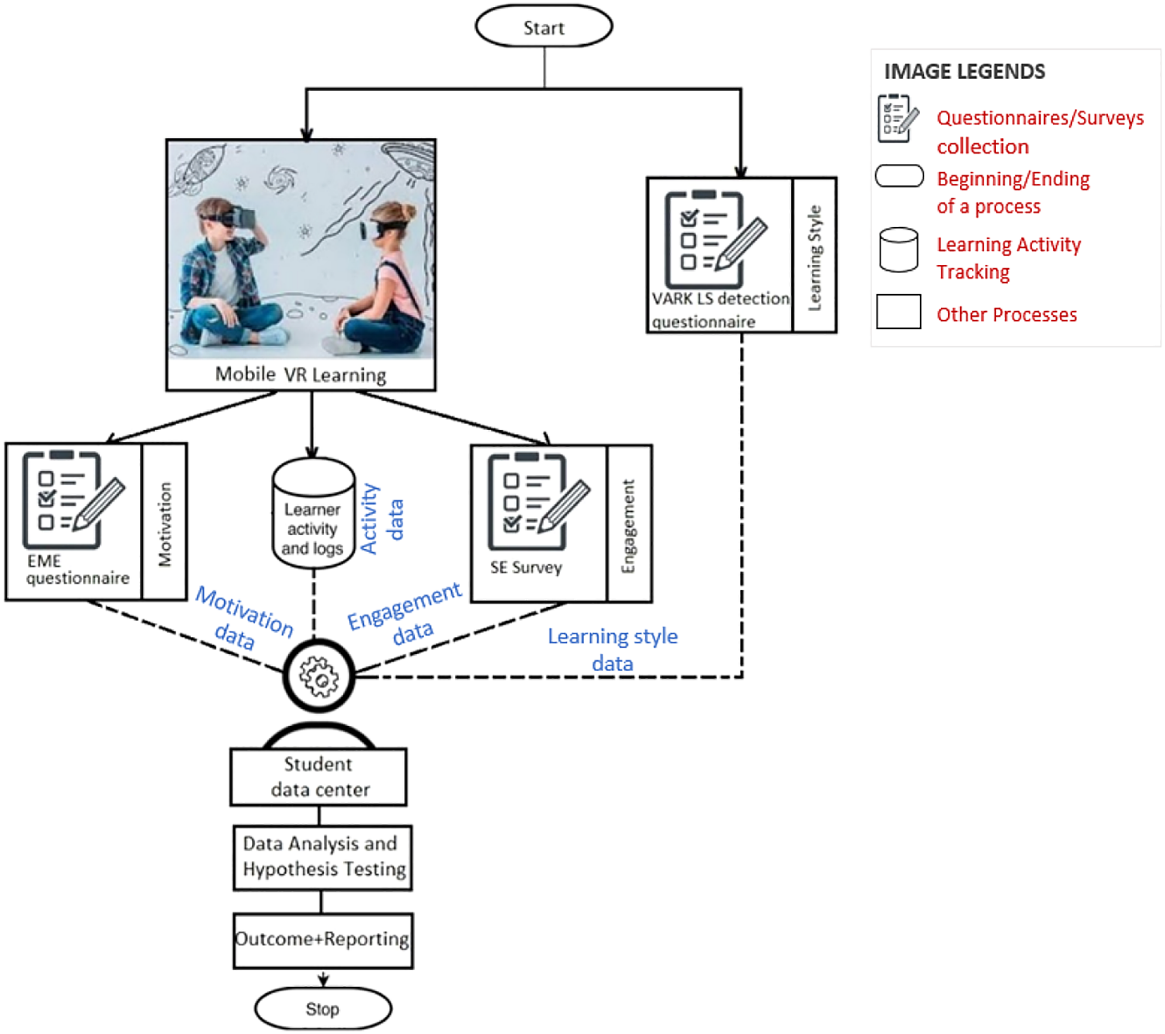
Process flow of the proposed framework.

The literature review shows that VR is swiftly becoming a household word, thanks to the recent boom of VR-compatible devices. Despite having a significant impact on student engagement and performance, mobile VR is greatly neglected in education. The proposed framework considers factors such as learning styles of learners and technology used to teach in order to measure the motivation and engagement of students belonging to the K-12 group ( Figure 3).

### Sample selection

A minimum cluster of 30 students from the same age group under the K-12 curriculum should be selected for the pilot study to be statistically significant. It is very important that the students be selected from a definite age group, preferably high schoolers (grade 9-12), and course, preferably STEM curriculum, as different age groups may produce different results. In addition, the study includes surveys in multiple levels; it’s desired that they be filled in with proper understanding of the questions being asked. High schoolers are young adults that fit the mentioned criteria. During the sample selection, diverse VARK learner types should be included in an even ratio to avoid a biased outcome and a healthy ratio of male to female should be considered to avoid skewing the results. This could be ensured with the “VARK LS Questionnaire” stage mentioned in Figure 3.

### Device selection

The most appropriate device for this study is Google Cardboard, as it is affordable, easy-to-build and has numerous vendors. It also supports both Android and iOS-based mobile devices. The VR devices (Google Cardboard) are to be acquired and distributed by the research team whereas the mobile devices can be the students’ own devices. A list of students’ existing mobile phones can be produced before initiating the test to ensure a smooth execution.

### Training and content design

Although most young learners are familiar with VR and can rapidly pick up technology usage or its trends, the teachers or instructors may need to get a better understanding of the possible uses and integration of the existing mobile VR software in the curriculum. Coming up with suitable educational content may require some training or professional help. This can be ensured by allowing time to the educators to explore the technology first and look though the manuals and tutorials available online. Then they can communicate their concerns or areas of difficulty and training/troubleshooting with the help of Google cardboard community can be arranged. For activity logs to be tracked, students will be taught a topic (in line with their study curriculum) using mobile VR technology which will be accompanied by a simple quiz to gauge how well the students understood the topic and how well they retained that information. This process should be repeated several times over the period of one semester to investigate the patterns over a decent time span. The design of the content should be appropriate for the age group, relevant to the topic, and have the ability to be supported by existing mobile VR software such as Expeditions, InCell VR, Titans of space, and Google Daydream. These VR software are some of the most well-established and widely used platforms for educational purposes with a variety of existing materials.

### Data collection

A mixed-method for data collection will be conducted to find the degree of engagement and motivation the learners learning in the mobile VR-assisted e-learning context.

A questionnaire method will be utilized as the first method to collect learner motivation and engagement-related data.

• Measuring motivation: the questionnaire developed by Vallerand *et al.* ^15^ known as EME [(Échelle de Motivation en Éducation (Measure of Motivation towards Education)], which comprises seven subscales measuring “three different kinds of intrinsic motivation and three different kinds of extrinsic motivation”. ^16^
• Measuring engagement: the Student Engagement (SE) survey developed by Ahlfeldt *et al.* ^23^ will be used that emphasizes the concepts of “cooperative learning, cognitive-level, and personal skills development, encompassing four, five, and five items respectively”. The three concepts are answerable on a four-point Likert scale, with 4 – very often, 3 – often, 2 – occasionally, and 1 – never. The aim of SE was to build an instrument that would be fast and easy to administer in class and which would measure student engagement.

A system log or activity log tracking will be used as the second method to collect data regarding engagement. Some of the system logs parameters proposed to be collected in this study are shown in Table 1.

**Table 1.** Variables to be tracked from activity logs.

Variable name	Units of measurement
Time spent on each content	Seconds
Time spent to complete a task	Seconds
Number of correct answers	Count
Number of wrong answers	Count
Number of attempts	Count
Number of questions asked	Count
Types of questions asked	Syntax

### Data analysis

The data collected through activity logs and questionnaires will be mapped to each individual learner correctly. Further visualization, analysis and investigation will be performed using Smart PLS, Python or R language to summarize the main characteristics of the data collected. Various methods of clustering, regression and correlation heatmaps will be applied to have an improved understanding of the relationships of the variables, detect patterns, spot anomalies, and test the hypothesis and other assumptions. Principal component analysis (PCA) will be explored to understand which attributes contribute most to the variance of each model. The Panda and Seaborn libraries in Python are very powerful in terms of data visualization and assessing correlation.

## Discussion

Many studies related to the use of mobile VR in the context of education have been carried out in recent years. These studies vary in terms of education level, course type, teaching environment, level of immersion, and more. There is however a lack of work that includes learning style frameworks in terms of using mobile VR for e-learning.

Pauschenwein *et al.* researched game-based learning elements that are economically priced and highly mobile VR systems with an enhanced feeling of immersion in the virtual environment and sound didactical scenarios. ^27^ The study focused on the method of making VR more affordable and widely used in practical studies but not so much on its potential in the field of e-learning. A pilot study carried out by Raya *et al.* compared mobile VR technology with conventional video content on a tablet device for teaching. The study was done on 56 high school learners looking into the effects of immersion and the induction of positive emotion while learning social science courses. The findings indicated that while delivering educational content, knowledge retention is heavily influenced by the immersive condition. Also, short-term participants of the study exhibited better retention when there were positive emotional induction and high immersion. Unlike our proposed study that integrates learning style element into the mobile VR and analyses its influences, this research focused on manipulating emotions, its impacts on knowledge retention, and high immersion as a potential technology in enhancing the influence of emotions. ^28^

Güray and Kısmet ^29^ proposed a model to integrate VR or AR (Augmented Reality) technologies in building construction education. Their proposed model highlighted the advantages of the creative use of VR/AR tools through integrating the Building Information Modelling tools in distance learning particularly during the Covid-19 pandemic conditions, but the VR/AR technology mentioned was not mobile supported and rather desktop based. One of the latest works done by Sprenger and Schwaninger ^30^ shed light on the technology acceptance of e-learning and mobile VR based on a three months usage. The voluntary participants were 94 students from a university in Northwestern Switzerland, studying the course “General Psychology 1”. The results suggested that the acceptance of mobile VR was very low compared to technologies like classroom response system, e-lectures, and classroom chat. Based on the theory of course alignment and examinations, the study revealed that students focused more on exam preparation rather than enjoying the process of learning. Apart from oversimplified VR content causing an underwhelming experience for some learners, the VR technology not having much relevance with the exam questions was also noted as one of the reasons for mobile VR having a low score on the spectrum of technology acceptance. The findings of the study re-emphasize the importance of selecting a proper learner age group and study level as well as an understanding of learner motivation and learning style while deploying a learning technology, which our conceptual model is greatly focusing on.

## Conclusion

Past research highlights the relationship between different learning styles and VR but there is very little research on the latter’s subbranch known as mobile VR and its usage in terms of e-learning. The conceptual framework is expected to bring the required focus on mobile VR and its potential usage in e-learning which is expected to greatly aid K-12 students belonging to more practical-oriented disciplines of study or those with a more visual or kinesthetic learning style. The framework allows an integration between e-learning and mobile VR supported by the principles of VARK learning styles. Its purpose is to enhance the motivation and engagement of e-learners by better understanding multiple learning styles in a mobile VR environment.

VR is considered one of the technology pillars of industry 4.0 or the fourth industrial revolution. ^13^ To introduce this fascinating innovation into classrooms, educators and facilitators should investigate the training needed to make it into a regularly used learning instrument. Although the idea of VR to some may appear as very advanced, in reality, it is quite similar to technology used in common social media apps, such as Instagram and Snapchat, home decor apps, such as Ikea Place, and gaming apps, such as Pokémon Go. Learning about mobile VR and its impact is a perfect place to start for educators who want to make their online teaching space VR-friendly.

Although the advantages of VR in teaching and learning are universal for a broad range of courses and learner types, the best integration approach differs from institution to institution as well as from class to class. Integrating mobile VR in terms of e-learning, on the other hand, is certainly achievable with the proper resources and knowledge on how VR will improve students' understanding and enthusiasm for learning. While the incorporation of VR technology in education is certain to enhance learning in almost every educational setting, the implementation approach does not demand to be radical. ^14^

There are challenges on traditional VR to pedagogical practice and theories such as cost, equipment usability, and fear of technology, ^11^ hence the education administrators need to jointly work together with the smartphone companies and VR product developers for its personalized, cost-effective implication at the post-pandemic stage to facilitate the process of e-learning.

## Data availability

No data is associated with this article.

## Grant information

This work was supported by TM R&D Grant [Grant ID MMUE/190022].
