# Supplementary material for: Post-pandemic e-learning: a pre-protocol to assess the impact of mobile VR on learner motivation and engagement for VARK learning styles
Source: F1000Res. 2022 May 19;10:1106. Originally published 2021 Nov 2. [Version 2] doi: 10.12688/f1000research.73311.2 (PMC9131843; doi:10.12688/f1000research.73311.2)
Supplement: Peer review history

# Peer review: Reviewer response for version 2

**DOI:** 10.5256/f1000research.134084.r138398

**Published:** 2022-05-20

**Authors:** Kenneth Y. T. Lim

**Affiliations:** 1 Office of Education Research, National Institute of Education, Singapore, Singapore

I thank the authorial team for taking the time and effort to understand the intent behind the suggestions from the preceding round of reviews.

 I am satisfied that you have addressed the concerns raised to the best of your ability.

 As such, i have no further comments to make.

Is the study design appropriate for the research question?

Yes

Is the rationale for, and objectives of, the study clearly described?

Yes

Are sufficient details of the methods provided to allow replication by others?

Yes

Are the datasets clearly presented in a useable and accessible format?

Not applicable

Reviewer Expertise:

the learning sciences, learning with VR / AR / XR, learning with mobile technology, learning during the COVID-19 pandemic, curriculum design and development

I confirm that I have read this submission and believe that I have an appropriate level of expertise to confirm that it is of an acceptable scientific standard.

# Peer review: Reviewer response for version 1

**DOI:** 10.5256/f1000research.76954.r122923

**Published:** 2022-02-14

**Authors:** Kenneth Y. T. Lim

**Affiliations:** 1 Office of Education Research, National Institute of Education, Singapore, Singapore

This manuscript is a study protocol and will be reviewed as such.

 The rationale for the protocol is justified in terms of current socio-economic contexts and the review of literature emerges from the rationale. I also find coherence between the review of literature, research questions, and the proposed methodology.

 The protocol proposed essentially seeks to investigate learner engagement and motivation with mobile VR from a theoretically-grounded perspective in the context of (a) the on-going pandemic and (b) the high prevalence of mobile technological penetration in relatively less economically developed countries.

 In this regard, I offer only the following suggestions for improvement:

 (a) In the review of literature, you refer to VARK variously as a "model" and a "tool". Please be consistent in how you perceive and refer to it.

 (b) I feel that such the outcomes of such a study - once carried out - would be only as strong as the curricular materials developed for the chosen technology (Google Cardboard). I would therefore like the authors to elaborate more on this, and not just content themselves to the three sentences beginning respectively with "Coming up with suitable...", "This can be ensured by...", and "Then they can communicate their concerns...".

Is the study design appropriate for the research question?

Yes

Is the rationale for, and objectives of, the study clearly described?

Yes

Are sufficient details of the methods provided to allow replication by others?

Yes

Are the datasets clearly presented in a useable and accessible format?

Not applicable

Reviewer Expertise:

the learning sciences, learning with VR / AR / XR, learning with mobile technology, learning during the COVID-19 pandemic, curriculum design and development

I confirm that I have read this submission and believe that I have an appropriate level of expertise to confirm that it is of an acceptable scientific standard, however I have significant reservations, as outlined above.

# Response

**Published:** 2022-05-15

**Authors:** Shahida Manzoor

**Affiliations:** MULTIMEDIA UNIVERSITY, Malaysia

Thank you, Dr. Kenneth Y. T. Lim for taking out the time to review the article. The suggestions/comments have been addressed as follows:

 a) *Changed* VARK framework/tool>model for consistency.

 b) The current study is to be carried out by utilizing existing VR software and study curriculum to understand its (current software's + curriculums') influence better and aims to later aid in the development of curriculum (new) based on the findings. *Suggestions made for this article have been added in the Future Work (section).*

# Peer review: Reviewer response for version 1

**DOI:** 10.5256/f1000research.76954.r98843

**Published:** 2022-02-03

**Authors:** Tunku Badariah Tunku Ahmad, Mohamad Sahari Nordin

**Affiliations:** 1 Department of Curriculum & Instruction, Kulliyyah of Education, International Islamic University Malaysia, Kuala Lumpur, Malaysia; 2 International Islamic University Malaysia, Selangor, Malaysia

**Summary of Feedback**

 1. The researchers/authors need to align the title with the research objectives and the constructs of interest (learner engagement and motivation). How do the authors define engagement and motivation?

 2. The present hypotheses appear to contradict one another, and should instead appear as follows:

**H1**: The effect of the VR intervention on learner engagement will vary significantly by learning style.

**H2**: The effect of the VR intervention on learner motivation will vary significantly by learning style.

 3. VARK learning styles are the moderator variable because the research aims to examine if the VR effects on learner engagement and motivation would vary significantly by the respondents' style of learning (i.e. whether they have a visual, auditory, reader/writer and kinesthetic learning style). In other words, you may find the results as such: The VR application increased students' learning engagement significantly for all groups, but its impact on students with visual and auditory learning styles was significantly greater, as an example, at Cohen's d = 0.87 and d = 0.91, respectively.

 4. A sample of 30 respondents is too small to test the hypotheses or run PCA/SEM on the data. The general rule is to have 5 to 10 respondents for every questionnaire item. For example, if the questionnaires have a total of 20 items, so the minimum sample needed is 20 x 5 = 100 (at least) or 20 x 10 = 200 respondents. PCA will not run if the minimum sample requirement is not met.

 5. Revisit the techniques of data analysis. SEM appears to be most appropriate for addressing the research objectives (while treating VARK as the moderator variable), on the condition that the sample size is large enough.

 See Annotated Manuscript for Further Remarks. https://f1000researchdata.s3.amazonaws.com/supplementary/73311/8047b5da-7165-4c21-9a47-177351dd26a9.pdf.

Is the study design appropriate for the research question?

Yes

Is the rationale for, and objectives of, the study clearly described?

Partly

Are sufficient details of the methods provided to allow replication by others?

Partly

Are the datasets clearly presented in a useable and accessible format?

Not applicable

Reviewer Expertise:

Pedagogical Studies, Web 2.0 tools, technology acceptance

We confirm that we have read this submission and believe that we have an appropriate level of expertise to confirm that it is of an acceptable scientific standard, however we have significant reservations, as outlined above.

# Peer review: Reviewer response for version 1

**DOI:** 10.5256/f1000research.76954.r115777

**Published:** 2022-02-02

**Authors:** Aslina Baharum

**Affiliations:** 1 Faculty of Computing and Informatics, Universiti Malaysia Sabah, Kota Kinabalu, Malaysia

This paper proposed a pre-protocol to investigate learner motivation and levels of engagement for e-learning with smartphone-integrated VR, based on their VARK (Visual, Auditory, Read/Write, Kinesthetic) learning styles. However, the paper needs to be improved for better presentation:

1. The findings of the paper should be added to the abstract.
2. Furthermore, no method is given. Authors should tell the reader how and what method(s) were used to get their findings. What was the proposed methodology, the authors should also give examples, and explain what systematic review has been used etc.
3. No data has been collected since this paper only provides the proposed method, but using method such as systematic review, data from the Literature Review can be extracted. Justification for the proposed method should included as well.
4. In the conclusion, authors should suggest future works they recommend for further examination, for example, the process for verifying and validating the proposed instrument or method, or data collection, based on the limitations of the study. 

Is the study design appropriate for the research question?

Yes

Is the rationale for, and objectives of, the study clearly described?

Yes

Are sufficient details of the methods provided to allow replication by others?

No

Are the datasets clearly presented in a useable and accessible format?

No

Reviewer Expertise:

User experience

I confirm that I have read this submission and believe that I have an appropriate level of expertise to confirm that it is of an acceptable scientific standard, however I have significant reservations, as outlined above.

# Response

**Published:** 2022-05-15

**Authors:** Shahida Manzoor

**Affiliations:** MULTIMEDIA UNIVERSITY, Malaysia

Thank you, Dr. Aslina Baharum for taking out the time to review the article. The suggestions/comments have been addressed as follows:

 1. As this is a *conceptual study/Study protocol*, only *what kind of results are expected* have been discussed and mentioned in the abstract.

 2. Narrative review has been used for this study, which synthesizes primary studies and explores this through description rather than statistics. (mentioned in the literature review)

 3. The methodology suggested for the proposed framework is derived and synthesized from the literature review. (mentioned in *discussion section *+ *literature review section*)

 4. *Future work* has been added (newly) in the conclusion section.
